# Monoclonal Antibodies and Antivirals against SARS-CoV-2 Reduce the Risk of Long COVID: A Retrospective Propensity Score-Matched Case–Control Study

**DOI:** 10.3390/biomedicines10123135

**Published:** 2022-12-05

**Authors:** Antonio Cimellaro, Desirée Addesi, Michela Cavallo, Francesco Spagnolo, Edoardo Suraci, Raffaella Cordaro, Ines Spinelli, Francesco Passafaro, Manuela Colosimo, Medea Pintaudi, Carmelo Pintaudi, on behalf of the CATAnzaro LOng COVID (CATALOCO) Study Group

**Affiliations:** 1 Internal Medicine Unit, Department of Medicine, “Pugliese-Ciaccio” Hospital of Catanzaro, Via Pio X n.83, 88100 Catanzaro, Italy; 2 Coordination of Special Unit of Continuity Care for Local Health Authority, Via Vinicio Cortese n.25, 88100 Catanzaro, Italy; 3 Microbiology and Virology Unit, Service Department, “Pugliese-Ciaccio” Hospital of Catanzaro, Via Pio X n.83, 88100 Catanzaro, Italy; 4 Neurophysiology and Neurobiology Unit, Department of Medicine, Fondazione Policlinico Universitario Campus Bio-Medico, Via Alvaro del Portillo n.200, 00128 Rome, Italy

**Keywords:** long COVID, post-COVID syndrome, COVID-19, SARS-CoV-2, monoclonal antibodies, antivirals

## Abstract

Long COVID is a complex condition affecting quality of life, with limited therapeutic options. We investigated the occurrence of long COVID in subjects receiving early therapy with monoclonal antibodies (mAbs) or antivirals to reduce the risk of COVID-19 progression. In this retrospective study we enrolled 737 adult patients (aged 65.16 ± 13.46; 361F), who experienced COVID-19 between January 2021 and March 2022. Antiviral or mAbs were administered to symptomatic patients who did not require oxygen therapy or hospital admission for SARS-CoV-2 infection, and who were at high risk of progression to severe disease, as identified by age > 65 years or the presence of comorbidities. Long COVID, defined as newly or persistent long-term symptoms 4 weeks after the onset of the acute illness, was reported in 204 cases (28%). Age (OR 1.03; *p* < 0.001), gender (OR 1.88; *p* < 0.001) and at least three comorbidities (OR 3.49; *p* = 0.049) were directly associated with long COVID; conversely, vaccination (OR 0.59; *p* = 0.005) and mAbs/antivirals (OR 0.44; *p* = 0.002) were independently associated with a reduced risk of long COVID. At a propensity-score-matched analysis, the mAbs/antivirals group had a significantly lower occurrence of long COVID in comparison with untreated controls (11% vs. 34%; *p* = 0.001). In conclusion, mAbs and antivirals administered against the progression of COVID-19 were associated with a reduced risk of long COVID.

## 1. Introduction

The coronavirus disease 2019 (COVID-19) pandemic is a worldwide outbreak of infectious disease caused by severe acute respiratory syndrome coronavirus 2 (SARS-CoV-2), with a broad clinical spectrum varying from asymptomatic manner to respiratory illness, multi-organ failure and fatal outcomes [1,2]. In response to this burden that led to over six million deaths since started [3], several scientific efforts have been made to advance vaccines and therapeutics into the clinical practice [4]. Among the various potential therapeutic interventions, monoclonal antibodies (mAbs) and oral antiviral drugs represent one of the most promising classes of molecules due to their specific activity against SARS-CoV-2, ability to support immune response and good safety profile [5,6]. Several studies support that early administration of mAbs or antivirals prevents disease progression and reduce hospitalization in subject at high risk for severe illness [7,8,9,10,11].

Despite advances in acute phase management, increasing evidence suggest that COVID-19 is associated with long-term sequelae—identified as post-COVID syndrome or long COVID—that negatively affect quality of life and represent a new challenge in the coming years [12]. Long COVID consists of a complex multisystem disease, occurring or persisting several weeks after recovery from acute infection with incidence estimated between 10 and 35% [13]. Patients affected by long COVID experience a wide, often vague and very subjective constellation of symptoms mostly characterized by shortness of breath, fatigue, palpitations, neuropsychiatric disorders and sensorial dysfunction. In such patients, long-term, clinically significant respiratory (e.g., post-infectious or post-embolic lung disease; residual respiratory failure), cardiovascular (e.g., dysrhythmia; heart failure) and gastrointestinal (e.g., diarrhea, constipation, abdominal pain) disorders occur [12,13,14]. 

To the current state of knowledge, pathophysiological mechanisms underlying the long COVID condition need to be elucidated and thus a therapeutic approach remains unclear. It is reasonable to speculate that preventing progression of SARS-CoV-2 infection by early therapy could be reduce the risk of long-term sequelae, but this hypothesis has never been tested. The aim of this retrospective research was to investigate the occurrence of long COVID in subjects at high risk of COVID-19 progression who received early therapy with mAbs or antivirals.

## 2. Materials and Methods

### 2.1. Study Population and Design

In this single-center retrospective study, we enrolled 737 consecutive patients from a large cohort of subjects affected by COVID-19, entrusted to the Special Unit of Continuity Care between January 2021 and March 2022 in the city of Catanzaro (Italy). All patients underwent collection of medical history and physical examination. Diagnosis of SARS-CoV-2 infection was detected by real-time reverse transcriptase–polymerase chain reaction (RT-PCR) on nasopharyngeal swab specimens. Clinical course was reported and evaluated by electronical medical records. Patients aged <18 years and those without available clinical data or who died were excluded. Main clinical outcome was the occurrence of long COVID: first, we examined frequencies and determinants of long COVID in the study population; then, we evaluated the occurrence of long COVID in subjects who received mAbs or antivirals against SARS-CoV-2 infection in comparison with who did not receive this early therapy, in a propensity-score-matched case–control study. The local ethics committee approved the protocol and informed written consent was obtained from all participants. All the investigations were performed according with the principles of Declaration of Helsinki.

### 2.2. Eligibility of Patients to Early Therapy with mAbs or Antivirals

Administration of mAbs or antivirals against SARS-CoV-2 was achieved in accordance with the criteria endorsed by Agenzia Italiana del Farmaco. Briefly, adult subjects were retained eligible to early therapy in the presence of the following criteria: (1) SARS-CoV-2 infection, confirmed by RT-PCR on nasopharyngeal swab specimens; (2) symptoms onset within 10 days for mAbs and 5 days for antivirals; (3) without hospital admission for COVID-19; (4) without oxygen therapy for COVID-19; (5) at high risk of progression to severe disease defined by the presence of at least one of the following factors: age >65 years, obesity, uncontrolled or complicated diabetes, chronic kidney disease or dialysis, primary or secondary immunodeficiency state, cardiovascular and cerebrovascular disease (including hypertension with organ damage), chronic respiratory disease or oxygen therapy for clinical reason other than COVID-19.

Among mAbs, the associations Bamlanivimab/Etesevimab (700/1400 mg) and Casirivimab/Imdevimab (1200/1200 mg) and the monotherapy Sotrovimab (500 mg) were administered as a single intravenous dose in a hospital setting, under clinical observation during and after inoculation, according to the drugs’ data sheets and modalities, as previously validated [7,8,9]. Among antivirals, Molnupiravir (800 mg BID) and Nirmatrelvir/Ritonavir (300/100 mg BID if renal filtrate > 60 mL/min; 150/100 mg BID if renal filtrate between 30 and 60 mL/min) were prescribed for oral intake for 5 days, according to the drugs’ data sheets and modalities, as previously validated [10,11]. All patients gave informed consent to administration, after being informed of the early therapy indications and potential adverse effects.

Patients were deemed ineligible for early therapy if any of the following criteria were present: refused administration; age < 18 years; ongoing pregnancy; history of allergic reactions to any component of the drugs; symptoms lasting more than 10 days and more than 5 days for mAbs and antivirals, respectively; clinical or radiological signs of COVID-19 pneumonia; oxygen therapy requirement due to COVID-19 progression; severe hepatic or renal impairment (filtrate < 30 mL/min) for antivirals.

### 2.3. Follow-Up and Definition of Long COVID

Follow-up was conducted firstly by phone survey 6 months after healing and then by visiting them at the dedicated “Long COVID” clinic referred to the Internal Medicine Unit of the “Pugliese-Ciaccio” Hospital of Catanzaro (Italy). All subjects were asked about clinical course, any hospitalizations, discharge summaries and diagnoses during and after SARS-CoV-2 infection. According to the literature [12], long COVID is defined as new and/or persistent symptoms and/or delayed or long-term complications beyond 4 weeks from the onset of the acute SARS-CoV-2 infection.

### 2.4. Statistical Analysis

Mean ± standard deviation for continuous variables, frequencies and percentages for categorical data were used to describe characteristics of the study population. To test for the differences among the observed clinical data, we used the unpaired Student’s *t*-test for continuous variables and the chi-square test for categorical variables, as appropriate. 

In order to identify factors associated with the occurrence of long COVID, a logistic regression analysis was performed, including in the multivariate model the variables that were significantly different at descriptive analysis or significantly associated with the outcome at univariate model, including age and gender. Data were expressed as odds ratio (OR), 95% confidence interval (CI) and *p* value. 

With the aim of minimizing bias due to potential confounding factors, the method of propensity score matching was used, assuming that an imbalance may exists in patients’ backgrounds between the group who received mAbs or antivirals and the group who did not. The propensity score for each patient was calculated as a probability using a logistic regression model, including all covariates that were considered clinically important and might have an impact on the outcome, with a one-to-one nearest-neighbor-matching algorithm at a caliper of 0.2 [15]. Variables included in this analysis were: gender; age; vaccination status; comorbidities; hospitalization. A two-sided *p* value < 0.05 was considered statistically significant. All analyses were performed using SPSS v. 25.0 (IBM, Armonk, NY, USA).

## 3. Results

### 3.1. Study Population and Occurrence of Long COVID

**Figure 1:**
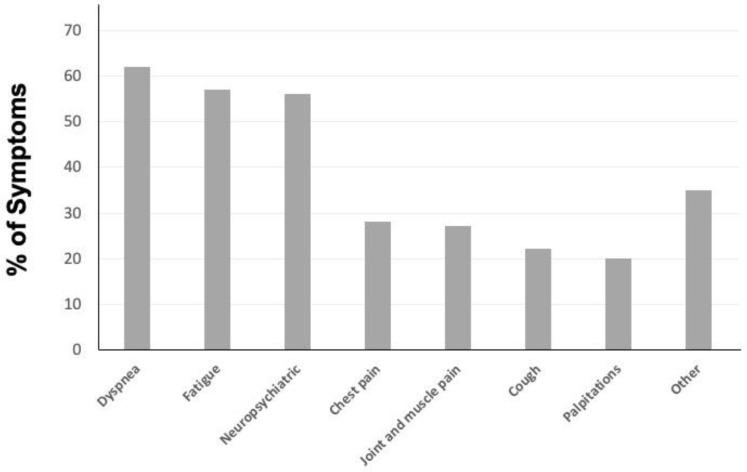
Percentage of symptoms reported in the long COVID group.

The demographic and clinical characteristics of the whole study population and according to presence or absence of long COVID are reported in Table 1. We analyzed 737 patients who suffered from COVID-19 aged 65.16 ± 13.46 (376 male and 361 female). Hypertension was the most represented comorbidity (571 cases, 78%), followed by diabetes (249 cases, 34%) and obesity (240 cases, 33%). Some patients were also affected by other cardiovascular diseases, such as coronary artery disease (167 cases, 23%), atrial fibrillation (131 cases, 18%) and heart failure (110 cases, 15%). Chronic respiratory and renal diseases were represented in a minority (114 cases equal to 16% and 51 cases equal to 7%, respectively). An immunodeficiency condition was reported in 12% of the whole population, mainly attributable to oncohematological disease. Of interest, one third of the population were affected by at least three comorbidities. With regard to vaccination status, a total of 504 cases (68%) were vaccinated and less than half of them with three doses (212 cases, 42%). A total of 135 patients received early therapy against SARS-CoV-2; in particular, 50 mAbs (5 Bamlanivimab/Etesevimab; 6 Casirivimab/Imdevimab; 39 Sotrovimab) and 85 antivirals (45 Molnupiravir; 40 Nirmatrelvir/Ritonavir) were administered. In relation to progression to severe disease, a total of 32 cases (4%) needed hospitalization; of these, only one patient underwent early therapy (antiviral Molnupiravir) and only eight patients were vaccinated (none of them were fully vaccinated with three doses). In Figure 1, we graphically represented the main symptoms reported by patients with long COVID in the study population.

With regard to the outcome of this study, we found 204 cases of post-COVID syndrome (28% of whole population). As reported in Table 1, patients with long COVID were significantly older (68.79 ± 12.08 vs. 63.77 ± 13.71; *p* < 0.001) and were mostly women (120 vs. 241; *p* = 0.001) when compared to those without. In addition, they were more commonly affected by hypertension (83% vs. 75%; *p* = 0.031) and diabetes (40% vs. 31%; *p* = 0.024) and had a greater clinical complexity as suggested by the percentage of those with at least three comorbidities (44% vs. 34%; *p* = 0.010). Hospital admission was more represented in the long COVID group (9% vs. 2%; *p* < 0.001). Interestingly, we observed that subjects who developed long COVID have less commonly received vaccination (59% vs. 72%; *p* = 0.001) and early therapy against SARS-CoV-2 with mAbs or antivirals (11% vs. 22%; *p* < 0.001). Regarding safety concerns, neither withdrawal nor severe or major adverse effects were observed in those who received early therapy. 

### 3.2. Determinants of Long COVID

To identify the main determinants of long COVID occurrence we performed a logistic regression analysis (Table 2). At univariate model, we observed that progression to long COVID was directly associated with age (OR 1.03; *p* < 0.001), female gender (OR 1.73; *p* = 0.001), hospital admission (OR 4.11; *p* < 0.001) and comorbidities (if 2 OR 3.58; *p* = 0.041; if ≥3 OR 4.64; *p* = 0.013). Conversely, vaccination (OR 0.57; *p* = 0.001) and early therapy with mAbs or antivirals (OR 0.42; *p* = 0.001) were inversely related with incidence of long COVID. Using the multivariate model including all the covariates previously tested, we observed that age, gender and at least three comorbidities remained significantly associated with long COVID occurrence; of interest, vaccination (OR 0.59; *p* = 0.005) and early therapy with mAbs/antivirals (OR 0.44; *p* = 0.002) were confirmed as variables independently associated with a reduced risk of long COVID.

### 3.3. Propensity-Score-Matched Case-Control Comparison

To further investigate the role of early therapy with mAbs or antivirals in the incidence of post-COVID condition, we performed a propensity-score-matched analysis to eliminate the risk of potential bias and confounding factors [15]. In the model, we included the variables that were found to be significantly related to long COVID occurrence through logistic regression analysis. We obtained a sample of 178 patients which were propensity-score-matched by age, gender, vaccination status, hospital admission and comorbidities, comprising 89 cases who received (mAbs/antivirals +; treated) and 89 controls who did not receive (mAbs/antivirals −; untreated) early therapy. In Table 3, demographic and clinical characteristics of the propensity-score-matched case (treated) and control (untreated) groups are shown.

**Figure 2:**
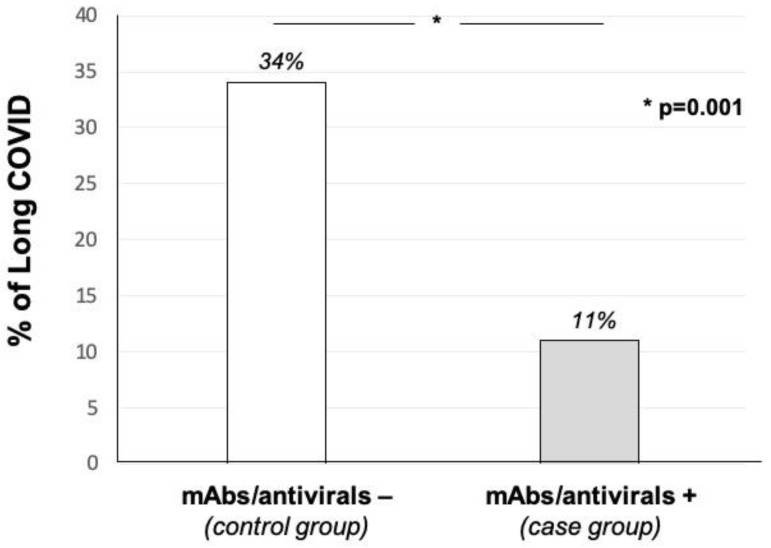
Percentages of long COVID according to propensity-score-matched case–control groups.

As shown in Figure 2, we observed a significantly lower occurrence of long COVID in the group who received mAbs or antivirals (10 cases equal to 11% vs. 30 cases equal to 34%; *p* = 0.001), supporting the hypothesis that early therapy could have a protective effect against post-COVID sequelae.

## 4. Discussion

In this retrospective, propensity-score-matched case–control study, we have demonstrated for the first time that early therapy with mAbs or antivirals against acute SARS-CoV-2 infection is able to reduce the risk of occurrence of long COVID syndrome. Long COVID represents a broad and very heterogeneous clinical entity, with pathophysiological mechanisms which have not been fully elucidated and, consequently, have a lack of established therapies or prophylaxis [12,13,14,15,16]. In this large Italian cohort of COVID-19 patients, we reported an occurrence of long COVID in approximately 28% of cases, almost confirming most epidemiological available data [12,13,14,15,16]. Moreover, subjects affected by long COVID in our study population experienced a wide spectrum of symptoms, mainly dyspnea, fatigue and neuropsychiatric concerns that negatively affected quality of life, as previously described [12,13,14,15,16]. Taken together, our results contribute to further highlighting the importance of a follow-up for those subjects who experienced SARS-CoV-2 infection, with the aim to better identify patients at risk. In this context, we have noticed that age and female gender were independently associated with long COVID occurrence, confirming what has been already observed [16,17]. Of interest, we also reported that patients who developed long COVID more commonly experienced a hospital admission during acute SARS-CoV-2 infection. In the literature, the relationship between the severity of COVID-19 and the incidence of long COVID is controversial: for example, some authors have documented that hospitalization for COVID-19 is not an independent risk factor for long COVID [18]. In our study population we reported that hospitalization was directly associated with a higher risk for long COVID occurrence at univariate logistic regression analysis, but not at multivariate model. Our explanation to that observation is based on a clinical issue: it is well established that patients affected by multiple comorbidities are at high risk for COVID-19 progression and, consequently, for hospitalization [19]. Assuming a biological plausibility, it is possible to speculate that there was a co-linearity between both variables—hospitalization and comorbidities—as suggested by the multivariate model in which patients with at least three comorbidities have a 3threefold higher risk of long COVID occurrence. Therefore, when stratifying risk for post-COVID sequelae, patients’ comorbidities and clinical complexities, rather than hospitalization, should be considered. On the other hand, increasing evidence suggest that the relationship between COVID-19 and comorbidities is bidirectional since SARS-CoV-2 infection can indeed exacerbate already-known chronic disease, or trigger new-onset illness [20,21,22,23,24,25,26,27]. This close relationship may be mostly attributed to a pathophysiological common soil between COVID-19 and comorbidities, characterized by endothelial damage, and increased inflammatory and thromboembolic burden [28,29]. Currently, given the lack of data about a treatment for clinically overt long COVID, a prophylactic strategy that prevents both the progression of COVID-19 and the occurrence of comorbidities’ complication, could be crucial. In this context, in our study population subjects with vaccination against SARS-CoV-2 had a reduced risk of long COVID, supporting the hypothesis that preventing disease progression could be important in preventing also long-term sequelae, as previously described [30]. Despite data confirming effectiveness of vaccination, we have demonstrated for the first time that mAbs and antivirals significantly reduced the risk of long COVID by 56%, with an excellent safety profile. Moreover, after elimination of potential bias and confounding factors in the propensity-score-matched case–control analysis, we observed a lower occurrence of long COVID in the treated group in comparison with the untreated control group (11% vs. 34%; *p* = 0.001). Taken together, these results are of significant clinical relevance because they could provide physicians with an additional treatment option for long COVID, based on the early administration of mAbs or antivirals, with the aim of reducing the risk of both disease progression and long-term sequelae.

### Study Limitations and Strengths

In the statistical inference analysis, we considered as dichotomous covariate together mAbs and antivirals (treated vs. untreated) to increase cases of subjects received early therapy, with the aim of testing the effect of early therapy on incidence of long COVID. We did not test mAbs or antivirals alone on the risk of long COVID due to reduced number of cases and possible low statistic power. Furthermore, qualitative and quantitative detection of patient-generated antibodies against SARS-CoV-2 was not available for all patients.

Despite the retrospective design of the study, the propensity-score-matched case–control approach reduced the potential bias in the analysis and improved the statistical power and biological plausibility of the observed data. However, these results should be confirmed in further research with a broader sample size and randomized, prospective design.

## 5. Conclusions

Early therapy with mAbs or antivirals in patients with SARS-CoV-2 infection at high risk for disease progression, in particular when affected by multiple comorbidities, was associated with reduced risk of long COVID.

## Figures and Tables

**Table 1:** Demographic and clinical characteristics of the study population.

Variables	All *n* = 737	Long COVID − *n* = 533	Long COVID + *n* = 204	*p*
Gender, M/F	376/361	292/241	84/120	0.001
Age, yrs	65.16 ± 13.46	63.77 ± 13.71	68.79 ± 12.08	<0.001
Hypertension, *n* (%)	571 (78)	402 (75)	169 (83)	0.031
CAD, *n* (%)	167 (23)	116 (22)	51 (25)	0.376
Atrial Fibrillation, *n* (%)	131(18)	93 (18)	38 (19)	0.747
Heart Failure, *n* (%)	110 (15)	82 (15)	28 (14)	0.644
Diabetes, *n* (%)	249 (34)	167 (31)	82 (40)	0.024
Obesity, *n* (%)	240 (33)	165 (31)	75 (37)	0.136
COPD/Asthma/ILD, *n* (%)	114 (16)	84 (16)	30 (15)	0.820
Immunodeficiency, *n* (%)	86 (12)	61 (11)	25 (12)	0.798
CKD, *n* (%)	51 (7)	36 (7)	15 (7)	0.744
Comorbidities, *n* (%)				0.010
0	31 (4)	28 (5)	3 (2)	
1	200 (27)	154 (29)	46 (23)	
2	238 (33)	172 (32)	66 (32)	
≥3	268 (36)	179 (34)	89 (44)	
Hospital Admission, *n* (%)	32 (4)	13 (2)	19 (9)	<0.001
Vaccination, *n* (%)	504 (68)	383 (72)	121 (59)	0.001
mAbs/antivirals, *n* (%)	135 (18)	114 (22)	21 (11)	<0.001

Long COVID −: absence of Long COVID; Long COVID +: presence of Long COVID; CAD: coronary artery disease; CKD: chronic kidney disease; COPD: chronic obstructive pulmonary disease; ILD: interstitial lung disease; mAbs: monoclonal antibodies.

**Table 2:** Logistic regression analysis for long COVID occurrence.

	Univariate Model		Multivariate Model	
Variables	Odds Ratio (95% CI)	*p*	Odds Ratio (95% CI)	*p*
Age, yrs	1.03 (1.02–1.05)	<0.001	1.03 (1.02–1.05)	<0.001
Gender, female vs. male	1.73 (1.25–2.40)	0.001	1.88 (1.33–2.67)	<0.001
Hospital admission, yes vs. no	4.11 (1.99–8.48)	<0.001	-	-
Comorbidities				
1	-	-	-	-
2	3.58 (1.05–12.18)	0.041	-	-
≥3	4.64 (1.37–15.68)	0.013	3.49 (1.00–12.07)	0.049
Vaccination, yes vs. no	0.57 (0.41–0.80)	0.001	0.59 (0.41–0.85)	0.005
mAbs/antivirals, yes vs. no	0.42 (0.26–0.69)	0.001	0.44 (0.26–0.74)	0.002

CI: confidence interval; mAbs: monoclonal antibodies.

**Table 3:** Demographic and clinical characteristics of the propensity-score-matched case–control groups according to the administration of early therapy (untreated vs. treated).

Variables	Control (Untreated) *n* = 89	Case (Treated) *n* = 89	*p*
Gender, M/F	40/49	39/50	0.880
Age, yrs	65.29 ± 12.59	65.69 ± 12.54	0.832
Hospital admission, *n* (%)	3 (4)	3 (4)	0.999
Comorbidities ≥ 3, *n* (%)	17 (19)	17 (19)	0.999
Vaccination, *n* (%)	61 (69)	61 (69)	0.999

## Data Availability

Data presented in this study are available on request from the corresponding author.

## Appendix A

All the Investigators and co-authors, in the Appendix below, must be tagged as collaborators and should appear in the PubMed record for this article.

APPENDIX: Investigators and co-authors of the CATALOCO (CAtanzaro LOng COVID) Study Group are as follows: Steering Committee: Antonio Cimellaro (Chair) (“Pugliese-Ciaccio” Hospital of Catanzaro, Italy), Carmelo Pintaudi (co-chair) (“Pugliese-Ciaccio” Hospital of Catanzaro, Italy), Desiree Addesi (“Pugliese-Ciaccio” Hospital of Catanzaro, Italy), Michela Cavallo (“Pugliese-Ciaccio” Hospital of Catanzaro, Italy), Francesco Spagnolo (“Pugliese-Ciaccio” Hospital of Catanzaro, Italy), Edoardo Suraci (“Pugliese-Ciaccio” Hospital of Catanzaro, Italy), Francesco Passafaro (Coordinator of Special Unit of Assistance Continuity of Catanzaro, Italy), Ilario Lazzaro (Healthcare Director for Local Health Authority of Catanzaro, Italy), Maria Rosaria Maione (Primary Care Department Director for Local Health Authority of Catanzaro, Italy). Investigators and collaborators: Maria Assunta (“Pugliese-Ciaccio” Hospital of Catanzaro, Italy), Giuseppe Bevacqua (“Pugliese-Ciaccio” Hospital of Catanzaro, Italy), Sara Carè (“Pugliese-Ciaccio” Hospital of Catanzaro, Italy), Anna Codispoti (“Pugliese-Ciaccio” Hospital of Catanzaro, Italy), Raffaella Cordaro (“Pugliese-Ciaccio” Hospital of Catanzaro, Italy), Santo Conte (“Pugliese-Ciaccio” Hospital of Catanzaro, Italy), Paola Costa (“Pugliese-Ciaccio” Hospital of Catanzaro, Italy), Francesca De Fazio (“Pugliese-Ciaccio” Hospital of Catanzaro, Italy), Vincenzo De Gennaro (“Pugliese-Ciaccio” Hospital of Catanzaro, Italy), Marcella Fratto (“Pugliese-Ciaccio” Hospital of Catanzaro, Italy), Federica Grandinetti (“Pugliese-Ciaccio” Hospital of Catanzaro, Italy), Andrea Greco (“Pugliese-Ciaccio” Hospital of Catanzaro, Italy), Antonio Imbesi (“Gaetano Martino” Hospital of Messina, Italy), Ilaria Levato (“Pugliese-Ciaccio” Hospital of Catanzaro, Italy), Daria Macrillò (“Pugliese-Ciaccio” Hospital of Catanzaro, Italy), Danilo Maiolo (“Pugliese-Ciaccio” Hospital of Catanzaro, Italy), Marco Mandaliti (“Pugliese-Ciaccio” Hospital of Catanzaro, Italy), Tania Mastroianni (“Pugliese-Ciaccio” Hospital of Catanzaro, Italy), Cristina Mauro (“Pugliese-Ciaccio” Hospital of Catanzaro, Italy), Grazia Multari (“Pugliese-Ciaccio” Hospital of Catanzaro, Italy), Francesca Piera Presentino (“Pugliese-Ciaccio” Hospital of Catanzaro, Italy), Salvatore Pignanelli (“Pugliese-Ciaccio” Hospital of Catanzaro, Italy), Federica Pignola (“Pugliese-Ciaccio” Hospital of Catanzaro, Italy), Rosetta Porco (“Pugliese-Ciaccio” Hospital of Catanzaro, Italy), Francesco Pullia (“Pugliese-Ciaccio” Hospital of Catanzaro, Italy), Annamaria Rotundo (“Pugliese-Ciaccio” Hospital of Catanzaro, Italy), Laura Rubino (“Pugliese-Ciaccio” Hospital of Catanzaro, Italy), Mariateresa Santo (“Pugliese-Ciaccio” Hospital of Catanzaro, Italy), Davide Saragnese (“Pugliese-Ciaccio” Hospital of Catanzaro, Italy), Claudio Servello (“Pugliese-Ciaccio” Hospital of Catanzaro, Italy), Sandra Sinopoli (“Pugliese-Ciaccio” Hospital of Catanzaro, Italy), Ines Spinelli (“Pugliese-Ciaccio” Hospital of Catanzaro, Italy), Francesca Tallarico (“Pugliese-Ciaccio” Hospital of Catanzaro, Italy), Alessia Vuono (“Pugliese-Ciaccio” Hospital of Catanzaro, Italy).
